# Three-dimensional radiomics of triple-negative breast cancer: Prediction of systemic recurrence

**DOI:** 10.1038/s41598-020-59923-2

**Published:** 2020-02-19

**Authors:** Jieun Koh, Eunjung Lee, Kyunghwa Han, Sujeong Kim, Dong-kyu Kim, Jin Young Kwak, Jung Hyun Yoon, Hee Jung Moon

**Affiliations:** 1 Department of Radiology, CHA Bundang Medical Center, CHA University, Seongnam, Korea; 2 Department of Computational Science and Engineering, Yonsei University, Seoul, Korea; 3 Department of Radiology, Research Institute of Radiological Science, Center for Clinical Imaging Data Science, Yonsei University, College of Medicine, Seoul, Korea; 4 Department of Biostatistics and Computing, Yonsei University Graduate School, Seoul, Korea; 5 Department of Radiology, Research Institute of Radiological Science, Severance Hospital, Yonsei University, College of Medicine, Seoul, Korea

**Keywords:** Image processing, Outcomes research

## Abstract

This paper evaluated 3-dimensional radiomics features of breast magnetic resonance imaging (MRI) as prognostic factors for predicting systemic recurrence in triple-negative breast cancer (TNBC) and validated the results with a different MRI scanner. The Rad score was generated from 3-dimensional radiomic features of MRI for 231 TNBCs (training set (GE scanner), n = 182; validation set (Philips scanner), n = 49). The Clinical and Rad models to predict systemic recurrence were built up and the models were externally validated. In the training set, the Rad score was significantly higher in the group with systemic recurrence (median, −8.430) than the group without (median, −9.873, *P* < 0.001). The C-index of the Rad model to predict systemic recurrence in the training set was 0.97, which was significantly higher than in the Clinical model (0.879; *P* = 0.009). When the models were externally validated, the C-index of the Rad model was 0.848, lower than the 0.939 of the Clinical model, although the difference was not statistically significant (*P* = 0.100). The Rad model for predicting systemic recurrence in TNBC showed a significantly higher C-index than the Clinical model. However, external validation with a different MRI scanner did not show the Rad model to be superior over the Clinical model.

## Introduction

Triple-negative breast cancer (TNBC) is a tumor subtype that lacks expression of the hormonal and HER2 receptors^1^, however, it is a genomically heterogeneous disease^2^. TNBC comprises 15% of all invasive breast cancers and tends to be detected when it is already large in size and high grade^1,3^. Systemic recurrence occurs more often in TNBC than non-TNBC and mostly within 5 years of the TNBC diagnosis, while non-TNBC shows constant recurrence risk during follow-up^4^. The overall 4-year survival rate of TNBC is 77.0%, which is lower than the overall survival rates of other subtypes which range from 82.7 to 92.5%^5^. Systemic recurrence of TNBC leads to poorer survival, and therefore, it is important to predict the systemic recurrence risk of TNBC to tailor treatment to the individual patient^4^. Tumor size, lymph node, androgen receptor, Ki-67, treatment options such as adjuvant chemotherapy, radiotherapy, and pathologic complete response (pCR) after neoadjuvant chemotherapy have been associated with TNBC prognosis^3,6–10^. Several studies have used breast magnetic resonance imaging (MRI) features to predict the prognosis of invasive breast cancer including TNBC^11–13^, and in other studies, peritumoral enhancement and perfusion parameters on MRI have been used for TNBC only^14,15^.

Radiomics shows promise for predicting cancer prognosis because it provides a comprehensive quantification of imaging phenotypes and allows an objective assessment of tumor phenotypes^16,17^. Tumors with higher heterogeneity are related with poorer survival, and because radiomics reflect tumor heterogeneity, radiomics of breast MRI have been reported as prognostic factors for invasive breast cancer^18–21^. To our knowledge, radiomics features using dynamic contrast-enhanced (DCE) MRI have not been evaluated to predict prognosis in TNBC, especially with 3-dimensional images from the whole tumor. One thing to note is that radiomics features of breast MRI are affected by the chosen imaging scanner^22^. Thus, radiomics features have to be validated with different scanners.

Therefore, the purpose of our study was to evaluate 3-dimensional radiomics features of breast MRI as prognostic factors for predicting systemic recurrence in TNBC. Furthermore, the results were externally validated with a different MRI scanner.

## Methods

### Study population

The Institutional Review Board approved this retrospective study and required neither patient approval nor informed consent for our review of patient images and medical records (Institutional review board, Severance Hospital Yonsei University College of Medicine, 4-2018-0520).

From January 2012 to December 2015, among 3,166 patients who underwent breast surgery due to breast cancer, 272 (8.6%) patients were confirmed with invasive TNBC. TNBC was defined when both estrogen receptors and progesterone receptors were <1% of tumor cell nuclei^28^ and also when HER2 staining scores were 1+ or 0, or 2+ with a fluorescence *in situ* hybridization amplification ratio of <2.0^29^. Forty-one patients were excluded because they had undergone MRI with different vendors for the training and validation sets (n = 19), had not undergone MRI before treatment (n = 8), had undergone MRI at an outside hospital (n = 6), had experienced problems when downloading the Digital Imaging and Communications in Medicine (DICOM) files (n = 5), had undergone vacuum-assisted biopsy (n = 2), or was a recurred case (n = 1). Finally, 231 TNBCs in 231 patients were included. Mean age of the patients was 51.1 ± 12.0 years (range, 23 to 88). The mean follow-up period was 43.2 ± 15.8 months.

### MRI acquisition

Breast MRI examinations were performed before any treatment using two 3-T MRI scanners (Discovery MR750w; GE Medical Systems, Milwaukee, Wisconsin, USA/Philips Achieva; Philips Medical Systems, Best, The Netherlands), and patients were imaged with the scanner used being decided arbitrarily according to the hospital’s daily schedule (182 for the GE scanner, 49 for the Philips scanner). Scans were performed with an 8-channel breast receiver coil with the patient in the prone position. Breast MRI consisted of T2-weighted axial images, fat-suppressed T2-weighted axial images, diffusion-weighted images, T1-weighted non-fat-suppressed pre-contrast images, 6 sets of 3D fat-suppressed DCE axial images, and T1-weighted fat-suppressed contrast-enhanced sagittal images. DCE-MRI was performed after injecting 20 mL of gadopentetate dimeglumine (Magnevist, Bayer HealthCare) over 15 seconds. Acquisition time of each DCE axial image was 79 seconds for the GE scanner (repetition time (TR)/echo time (TE), 6.2/1.3 ms; section thickness (ST), 3 mm; field-of-view (FOV), 32 cm) and 65 seconds for the Phillips scanner (TR/TE, 3.9/1.4 ms; ST, 1.5 mm; FOV, 32 cm). For each set of DCE axial images, subtraction images were generated.

### Radiomics analysis

The DICOM files of the second phase of fat-suppressed DCE-MRI for the GE scanner and second subtracted DCE-MRI images for the Philips scanner were downloaded to draw regions-of-interest (ROIs). One radiologist (H.J.M.) drew a ROI on every slice of MRI that demonstrated the index tumor along the tumor border while excluding normal breast parenchyma with the semi-automated process provided by the Medical Image Processing, Analysis, and Visualization (MIPAV) application (http://mipav.cit.nih.gov/index.php), and captured images within the new frame were saved. Radiomics analysis was performed on the ROI by one of the authors (E. L.). In order to reduce data redundancy and inconsistency, once a ROI was located, a box enclosing the ROI was extracted from the 3D image and the voxel values in the box were scaled to normalize the intensity so that the data read the same way across all images. Then, information on the exact position of the ROI boundary was gathered and applied to the same image without ROI segmentation to extract the ROI only image (Supplementary Fig. S1). This process was necessary to remove interference from drawing the ROI boundary with a colored marker when analyzing features. To normalize the images, we used min-max scaling so that the voxel values of each image were within a range from 0 to 255. As a pre-processing step, all images were normalized to have similar data distribution. The histogram analysis consisted of 14 features (Supplementary Fig. S2). Shape- and size-based features were made up of 8 features. Textural features were calculated from the gray-level co-occurrence matrix (GLCM) and gray-level run-length matrix (GLRLM). The 3-dimensional GLCM and GLRLM were calculated in 13 directions. GLCM features were analyzed for 22 parameters and GLRLM features were analyzed for 11 parameters. The 3-dimensional wavelet transform was then applied with 8 decompositions, by directional low-pass and high-pass filtering (X_LLL_, X_LHL,_ X_LLH,_ X_HLL,_ X_LHH,_ X_HLH,_ X_HHL_ and X_HHH_). Matlab R2018a was used to generate the corresponding values of each parameter (The MathWorks, Inc. Natick, MA, US).

### Establishing the Rad score

The total number of radiomic features was 3995. We used the least absolute shrinkage and selection operator (LASSO) with 10-fold cross-validation to identify the optimal number of features to predict systemic recurrence in the training set. Cross-validation was repeated 100 times to minimize bias from random partition and coefficients of the features were calculated. Relative standard deviation (standard deviation/mean) was calculated from the coefficients to select features that predicted systemic recurrence. The equation for the Rad score is as follows.

Rad score = M_1_X_1_ + M_2_X_2_ + .. + M_n_X_n_ (M_n_: coefficients of regression, X_n_: selected features)

### Data analysis

Clinical and pathologic data were reviewed. Patient age, neoadjuvant chemotherapy, surgery type, adjuvant chemotherapy, and radiotherapy were recorded. On the pathologic report, presence of pCR after neoadjuvant chemotherapy, pathologic invasive tumor size, nuclear grade, histologic grade, lymphovascular invasion, and the number of metastatic axillary lymph nodes after surgery were recorded. Interval from initial diagnosis to systemic recurrence or the date of the last follow-up was recorded. Patients were assigned to the training and validation sets according to the MRI scanner used. Patients who underwent MRI examinations with the GE scanner were classified as the training set (n = 182) and those who underwent MRI with the Philips scanner were classified as the validation set (n = 49), respectively.

### Statistical analysis

Continuous variables of the training and validation sets were compared with the Mann-Whitney U test or independent two-sample t-test, and categorical variables were compared with Fisher’s exact test or the chi-square test, respectively. The Rad score was compared according to systemic recurrence with the Mann-Whitney U test. Univariable Cox proportional hazard regression was performed for the features predicting recurrence. Multivariable Cox proportional hazard regression was performed with the best subset selection method over 6 models to select the appropriate combination of features for external validation because the number of variables was larger than the number of events (Supplementary Table S3). For all 6 models, the Rad score was compared one-on-one with six variables which were significantly associated with systemic recurrence on univariable analysis. The concordance-index (C-index) for predicting systemic recurrence was calculated in the training set to evaluate the discriminative ability of each model^30^. From the six examined models, features that showed a high C-index for predicting systemic recurrence in the training set were selected to create the Clinical and Rad models. The Rad score consistently remained significant for the 6 models examined. We excluded pCR and histologic grade, because the data was completely separable and also showed low C-indexes. Moreover, pCR could be replaced by invasive tumor size on pathology and histologic grade was only significant between grade 1 and not available categories which were derived from pCR cases on the univariable analysis. The Clinical model was built up with only clinicopathologic variables. The Rad model was composed with the Rad score added to the Clinical model. External validation was performed with the features selected through multivariable analysis, and the C-index was calculated. We compared the C-indexes of the Clinical and Rad models. Statistical analyses were performed by R Statistical Software (version 3.5.1.; R Foundation for Statistical Computing, Vienna, Austria).

## Results

### Patients’ characteristics and recurrence events

The clinical and pathologic characteristics were compared between the training and validation sets and the results are presented in Table 1. Interval from initial diagnosis to systemic recurrence or the date of the last follow-up was significantly different between the two groups (*P* < 0.001). All other characteristics were not significantly different. Systemic recurrence was observed in 22 (9.5%) cases (training set, n = 19; validation set, n = 3). The median time to recurrence was 18.8 months (mean, 22.9 months; range, 7.0 to 61.6 months). The organs with tumor recurrence were the lungs (n = 14), liver (n = 10), bones (n = 8), distant lymph nodes (n = 8), brain (n = 7), peritoneum (n = 1), and adrenal gland (n = 1) with 19 cases showed multiple sites of recurrence.

**Table 1:** Comparison of clinical and pathologic characteristics between the training and validation sets.

Variables	Training set (n = 182)	Validation set (n = 49)	*P* value
Age, mean ± standard deviation	50.84 ± 12.35	51.88 ± 10.53	0.556
Neoadjuvant chemotherapy, n (%)			>0.999
Yes	70 (38.46)	19 (38.78)	
No	112 (61.54)	30 (61.22)	
pCR, n (%)			0.913
Not applicable	112 (61.54)	30 (61.22)	
Yes	33 (18.13)	10 (20.41)	
No	37 (20.33)	9 (18.37)	
Pathologic invasive cancer size, median (IQR)	14 (5.25, 20)	15 (7, 21)	0.503
Nuclear grade, n (%)			0.794
2	44 (24.18)	11 (22.45)	
3	125 (68.68)	36 (73.47)	
Not available	13 (7.14)	2 (4.08)	
Histologic grade, n (%)			0.896
1	2 (1.1)	0 (0)	
2	49 (26.92)	14 (28.57)	
3	118 (64.84)	33 (67.35)	
Not available	13 (7.14)	2 (4.08)	
Lymphovascular invasion, n (%)			0.071
Yes	11 (6.04)	7 (14.29)	
No	171 (93.96)	42 (85.71)	
Surgery type, n (%)			0.092
Brest conserving surgery	126 (69.23)	27 (55.1)	
Total mastectomy	56 (30.77)	22 (44.9)	
Number of metastatic axillary lymph nodes after surgery, median (IQR)	0 (0, 0)	0 (0, 0)	0.396
Adjuvant chemotherapy, n (%)			0.845
Yes	110 (60.44)	31 (63.27)	
No	72 (39.56)	18 (36.73)	
Radiotherapy, n (%)			0.135
Yes	156 (85.71)	37 (75.51)	
No	26 (14.29)	12 (24.49)	
Systemic recurrence event, n (%)			0.583
Yes	19 (10.44)	3 (6.12)	
No	163 (89.56)	46 (93.88)	
Interval from initial diagnosis to systemic recurrence, median (IQR)	47.39 (36.73, 59.73)	33.03 (28, 36.87)	<0.001

pCR = pathologic complete response, IQR = interquartile range.

### Radiomic features and Rad score

**Figure 1:**
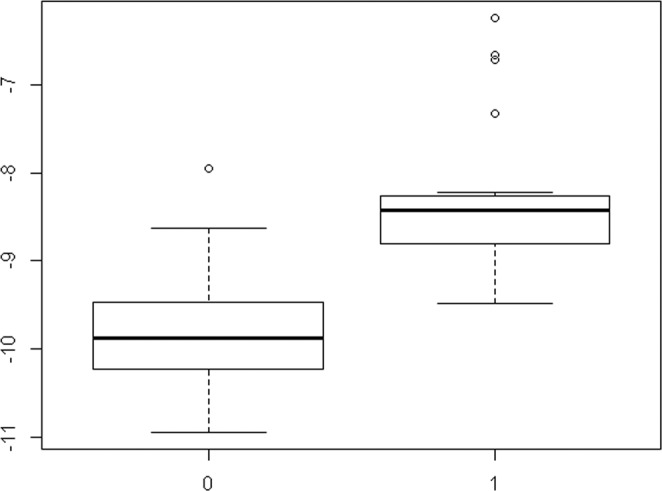
Distribution of the Rad score according to systemic recurrence. The Rad score was significantly higher in the group with systemic recurrence (Label 1, median, −8.430; interquartile range (IQR), −8.800 to −8.259) than the group without (Label 0, median, −9.873; IQR, −10.226 to −9.468, *P* < 0.001).

The mean number of features selected from 100 repeats of the 10-fold cross-validation for LASSO was 32.23 (range 3 to 54). We chose mean number of 32 features to generate the Rad score (Table 2). Among them, 7 were selected from GLCM-related features, 24 from GLRLM-related features, and 1 from histogram analysis. The most frequently selected feature was difference entropy (n = 6). Twenty-five selected features were from wavelet decomposition. In the training set, the Rad score was significantly higher in the group with systemic recurrence (median, −8.430; interquartile range (IQR), −8.800 to −8.259) than the group without (median, −9.873; IQR, −10.226 to −9.468, *P* < 0.001) (Fig. 1).

**Table 2:** Selected 32 radiomics features of the Rad score.

Features	Angle	Wavelet	^*^Coefficient
IMC2	2	LLH	−12.727
DE	2	LHH	−1.37
SRLGLE	2	LLL	5.547
LRLGLE	12	HLL	6.093
LRLGLE	12		5.375
LRHGLE	1		0.002
LRHGLE	4		0.002
SRHGLE	4	LLH	−0.012
LRLGLE	13		4.556
LRE	4	LLL	0.043
LRLGLE	6	HLH	−7.015
LGLRE	6	HLL	6.238
SKEW	1		−0.204
RLN	11	HLL	−0.008
HGLRE	8	LHH	0.011
DE	2	HHH	0.371
HGLRE	7	HHH	0.007
DE	3		−0.354
DE	2	HLH	0.317
SRLGLE	6	LHL	4.157
DE	8	HLL	0.344
SRHGLE	5	HHH	0.008
RLN	7	HLL	0.004
GLN	13	HHH	0.021
DE	11	LLH	−0.203
SRLGLE	1	LLL	2.055
RP	9		0.878
SRLGLE	7	HLL	3.368
SRHGLE	7	HHL	−0.01
LRHGLE	7	LLL	−0.002
LRLGLE	12	LLL	−0.081
GLN	11	HLL	−0.017

IMC = informational measure of correlation, DE = difference entropy, SRLGLE = short run low gray-level emphasis, LRLGLE = long run low gray-level emphasis, LRHGLE = long run high gray-level emphasis, SRHGLE = short run high gray-level emphasis, LRE = long run emphasis, LGLRE = low gray-level run emphasis, SKEW = skewness, RLN = run-length non-uniformity, HGLRE = high gray-level run emphasis, GLN = gray-level non-uniformity, RP = run percentage, *Average of Coefficient was calculated from cross-validation.

### Clinical and Rad model for predicting systemic recurrence

On univariable analysis, pCR status, pathologic invasive cancer size, histologic grade, lymphovascular invasion, surgery type, number of metastatic axillary lymph nodes after surgery, and the Rad score were significantly associated with systemic recurrence (Table 3). Through the best subset methods, the Clinical model was built with pathologic invasive cancer size, lymphovascular invasion, surgery type, and number of metastatic axillary lymph nodes after surgery. The Clinical model for predicting systemic recurrence showed that lymphovascular invasion (hazard ratio (HR), 7.875; 95% confidence interval (CI), 2.679, 23.153; *P* < 0.001), surgery type (HR, 0.231; 95% CI, 0.078, 0.679; *P* = 0.008), and the number of metastatic axillary lymph nodes after surgery (HR, 1.06; 95% CI, 1.002, 1.121; *P* = 0.043) were statistically significant on multivariable analysis (Table 4). The C-index of the Rad score for predicting systemic recurrence in the training set was 0.964 (95% CI, 0.829, 1), which was significantly different from that of the Clinical model (0.879; 95% CI, 0.744, 1; *P* = 0.009). When the models were externally validated in the validation set, the C-index was 0.939 (95% CI, 0.604, 1) for the Clinical model and 0.765 (95% CI, 0.430, 1) for the Rad score. The C-index of the Rad score was significantly lower than that of the Clinical model (*P* = 0.001). The Rad model was composed with the Rad score added to the Clinical model. In the Rad model, lymphovascular invasion (HR, 4.414; 95% CI, 1.331, 14.637; *P* = 0.015) and the Rad score (HR, 39.302; 95% CI, 11.351, 136.078; *P* < 0.001) remained statistically significant. The C-index of the Rad model for predicting systemic recurrence in the training set was 0.97 (95% CI, 0.835, 1), which was significantly higher than the C-index of the Clinical model (0.879; 95% CI, 0.744, 1; *P* = 0.009).

**Table 3:** Univariable analysis of variables to predict systemic recurrence in TNBC.

Variables		Hazard ratio (95% CI)	*P* value
Age		0.978 (0.94, 1.017)	0.267
Neoadjuvant chemotherapy	Yes	1.656 (0.665, 4.121)	0.278
	No		
Adjuvant chemotherapy	Yes	0.617 (0.248, 1.53)	0.297
	No		
Radiotherapy	Yes	0.824 (0.239, 2.84)	0.76
	No		
^*^pCR	Yes	0.048 (0.003, 0.926)	<0.001
	Not applicable	0.244 (0.097, 0.613)	0.002
	No		
Pathologic invasive cancer size		1.049 (1.021, 1.078)	<0.001
^*^Nuclear grade	3	0.934 (0.337, 2.59)	0.892
	Not available	0.288 (0.014, 5.843)	0.317
	2		
^*^Histologic grade	3	0.113 (0.018, 0.694)	0.055
	2	0.188 (0.029, 1.221)	0.124
	Not available	0.042 (0.001, 1.244)	0.036
	1		
Lymphovascular invasion	Yes	13.2 (5.155, 33.804)	<0.001
	No		
Surgery type	Brest conserving surgery	0.167 (0.063, 0.443)	<0.001
	Total mastectomy		
Number of metastatic axillary lymph nodes after surgery		1.139 (1.084, 1.196)	<0.001
Rad score		23.322 (10.346, 52.575)	<0.001

TNBC = triple-negative breast cancer, CI = confidence interval, *Unbiased hazard ratio was estimated with Firth’s penalized maximum likelihood estimation method because the data was completely separable.

**Table 4:** Multivariable analysis of variables to predict systemic recurrence in TNBC.

Variables		Clinical model		Rad model	
		Hazard ratio (95% CI)	*P* value	Hazard ratio (95% CI)	*P* value
Pathologic invasive cancer size		1.014 (0.98, 1.049)	0.438	1.016 (0.968, 1.067)	0.516
Lymphovascular invasion	Yes	7.875 (2.679, 23.153)	<0.001	4.414 (1.331, 14.637)	0.015
	No				
Surgery type	Brest conserving surgery	0.231 (0.078, 0.679)	0.008	0.335 (0.084, 1.337)	0.121
	Total mastectomy				
Number of metastatic axillary lymph nodes after surgery		1.06 (1.002, 1.121)	0.043	1.002 (0.92, 1.091)	0.964
Rad score				39.302 (11.351, 136.078)	<0.001
C-index of the training set (95% CI)		0.879 (0.744, 1)		0.97 (0.835, 1)	
^*^P value		0.009			
C-index of the validation set (95% CI)		0.939 (0.604, 1)		0.848 (0.513, 1)	
^*^P value		0.100			

TNBC = triple-negative breast cancer, CI = confidence interval, ^*^P value was calculated by comparing the C-index between the Clinical and Rad models.

### External validation

When the models were externally validated in the validation set, the C-index was 0.939 (95% CI, 0.604, 1) for the Clinical model and 0.848 (95% CI, 0.513, 1) for the Rad model (Table 4). The C-index of the Rad model was lower than the Clinical model, even though there was no statistical difference (*P* = 0.100).

## Discussion

We tried to build up a model to predict systemic recurrence in TNBCs using the 3-dimensional radiomics features of pretreatment breast MRI. When the Rad score was combined with clinicopathologic variables (the Rad model), the new model was able to predict systemic recurrence better than the model comprised only with clinicopathologic variables (the Clinical model). However, the Rad model was not superior over the Clinical model in an external validation which used another MRI scanner with a different protocol.

The Rad score was extracted from a massive volume of radiomics features on MRI and was significantly higher in patients with systemic recurrence. Most features were selected from the GLCM and GLRLM, and the most frequently selected feature was the difference entropy of GLCM which was selected 6 times with different angles and wavelet combinations. Difference entropy is the measure of randomness and variability in neighborhood intensity value differences^23^. GLCM and GLRLM are calculated from the interaction between image pixels; thus, tumor heterogeneity can be better reflected with these parameters than histogram analysis or shape- and size-based features. Park *et al*. also found that GLCM had more valuable features for predicting the prognosis of invasive breast cancer although their study population was not TNBC-specific^18^.

Systemic recurrence occurred in 9.5% of our study population, which was within the range of a previous study^3,24,25^. The median interval to recurrence was 18.8 months and this finding was also seen in TNBC patients who relapsed and died within 5 years of diagnosis^4^. We created the Clinical model which was comprised of the pathologic invasive cancer size, lymphovascular invasion, surgery type, and number of metastatic axillary lymph nodes after surgery to predict systemic recurrence. The Clinical model in the training set showed a high C-index of 0.879 to predict systemic recurrence and the C-index of the Rad score itself was 0.964 (95% CI, 0.829, 1), which was significantly higher than the C-index of the Clinical model. The Rad model consisted of the Clinical model plus the Rad score. In the training set, the Rad model showed a significantly higher C-index of 0.97 than the Clinical model (*P* = 0.009) for predicting systemic recurrence in TNBC. While there have been several radiomics studies on invasive breast cancers and prognosis, they have not been TNBC-specific. Kim and colleagues evaluated entropy and uniformity only and they associated higher entropy on T2-weighted images and lower entropy on contrast-enhanced T1-weighted subtraction images with poorer recurrence-free survival in invasive ductal carcinoma^20^. Yamamoto and colleagues investigated only eight radiomics features and an enhancing rim fraction was related with metastasis-free survival^19^. Li and colleagues showed that there was a relationship between radiomics and multigene assays, but their study did not evaluate recurrence nor survival^21^. Park and colleagues studied four radiomics features and reported that the combination of radiomics and clinicopathologic features could predict disease-free survival^18^. The strength of our study is that we analyzed 3-dimensional images from the whole tumor with 3995 derived radiomics features, and thereby took full advantage of the massive volume of radiomic features. The Rad model which added the Rad score to the Clinical model showed better performance than the Clinical model to predict systemic recurrence in TNBC patients.

We validated the Rad model with a different MRI scanner and different protocol. The Rad model of the validation set also showed a high C-index of 0.848. However, the value of 0.848 was lower than the 0.939 of the Clinical model even though there was no statistical difference. In a study on prediction of local tumor control after radio-chemotherapy for head and neck cancer, computed tomography (CT) radiomics were investigated and the results were validated with a different CT model and protocols. A past study experienced lower performance with the clinical plus radiomics models in the validation set than the training set, and they assumed that different CT protocols or parameters would not affect the results^26^. Another prior study used positron emission tomography–computed tomography radiomics with different scanners to predict the recurrence of cervix cancer and a higher C-index was observed in the training set than in the validation set^27^. We also used different MRI scanners with the GE scanner being used for the training set and the Philips scanner being used for the validation set, and different protocols were used for the fat-suppressed enhanced images of the training set and subtracted images of the validation set. The Rad model showed lower performance in the validation set. We could not find studies on MRI radiomics that had validated their findings with a different scanner; however, according to an experimental study, the type of MRI machine used and slice thickness can affect the radiomics data^22^. We assumed that a difference between the two scanners might have caused inconsistent results in the training set. The time of acquisition for the second phase of DCE-MRI was different between the two scanners. ROIs were drawn on the fat-suppressed native images for the training set, but were drawn on the subtracted images for the validation set. Therefore, we had inconsistent results between the training and validation sets.

We acknowledge that there were several limitations in this study. First, the number of included patients was relatively small and the study was performed retrospectively. However, TNBC only comprises a small portion of invasive breast cancer. Second, this study was performed in a single institution. External validation at other institutions is necessary although we validated our results with a different MRI scanner and protocol to minimize overfitting. Third, we only analyzed the second phase of the DCE T1-weighted images. While it would be ideal to include all sequences of the dynamic images for evaluation, we chose one sequence from the DCE-MRI to simplify the methods and results, as the second phase is the most important phase in breast cancer evaluation. Early enhancement is a representative character of breast cancer which is why we chose this sequence. T2-weighted images were valuable in a previous radiomics study^18,20^ and, therefore, this study should be followed with further studies that include the delayed phase of DCE T1-weighted images or T2-weighted images. Fourth, ROIs were drawn by one radiologist, and inter- and intra-observer variabilities were not evaluated when drawing ROs for the radiomics analysis. However, we used the MIPAV application which drew ROIs along the tumor border that excluded the normal breast parenchyma with a semi-automated process. Thus, this would not have a great effect on the results. In addition, according to Park *et al*., drawing ROIs between the two observers showed good agreement^18^.

In conclusion, the Rad model for predicting systemic recurrence in TNBC showed a significantly higher C-index than the Clinical model. However, external validation with a different MRI scanner did not show the Rad model to be superior over the Clinical model.

## Supplementary information

Supplementary information.

## Data Availability

No datasets were generated or analysed during the current study.
