# Supplementary material for: Three-dimensional radiomics of triple-negative breast cancer: Prediction of systemic recurrence
Source: Sci Rep. 2020 Feb 19;10:2976. doi: 10.1038/s41598-020-59923-2 (PMC7031504; doi:10.1038/s41598-020-59923-2)
Supplement: Supplementary file 1 — Supplementary information. [file 41598_2020_59923_MOESM1_ESM.pdf]

Three-dimensional radiomics of triple-negative breast cancer: Prediction of systemic recurrence

Jieun Koh, MD[1], Eunjung Lee, PhD[2], Kyunghwa Han, PhD[3], Sujeong Kim, BS[4], Dong-kyu Kim, MD[5], Jin Young Kwak, MD, PhD[5], Jung Hyun Yoon, MD, PhD[5], Hee Jung Moon, MD, PhD[5*]

[1]Department of Radiology, CHA Bundang Medical Center, CHA University, Seongnam, Korea

[2]Department of Computational Science and Engineering, Yonsei University, Seoul, Korea

[3]Department of Radiology, Research Institute of Radiological Science, Center for Clinical Imaging Data Science, Yonsei University, College of Medicine, Seoul, Korea

[4]Department of Biostatistics and Computing, Yonsei University Graduate School, Seoul, Korea

[5]Department of Radiology, Research Institute of Radiological Science, Severance Hospital, Yonsei University, College of Medicine, Seoul, Korea

***Corresponding author: Hee Jung Moon**

Department of Radiology, Severance Hospital, Research Institute of Radiological Science, Yonsei University, College of Medicine

50 Yonsei-ro, Seodaemun-gu, 03722 Seoul, Korea

TEL: 82-2- 2228-7400, FAX: 82-2-393-3035

E-mail: artemis4u@yuhs.ac

Supplementary Figure S1

Figure S1-1. 2D slice images of a DICOM file

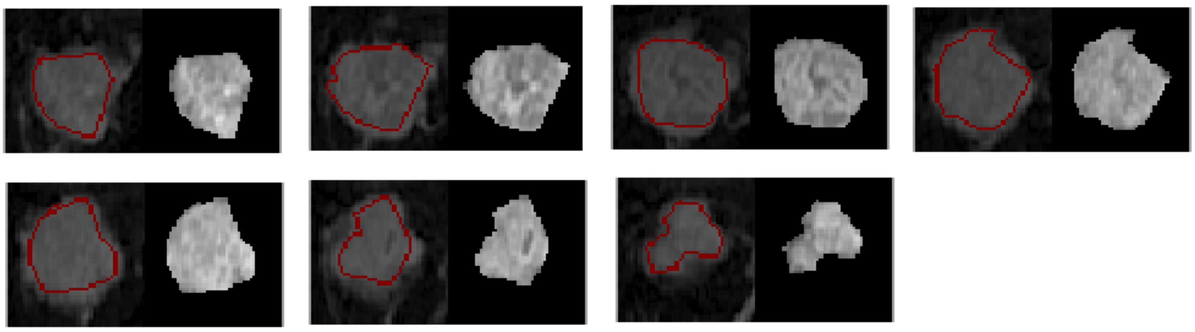

Figure S1-2. 3D reconstructed image based on slices of a DICOM file

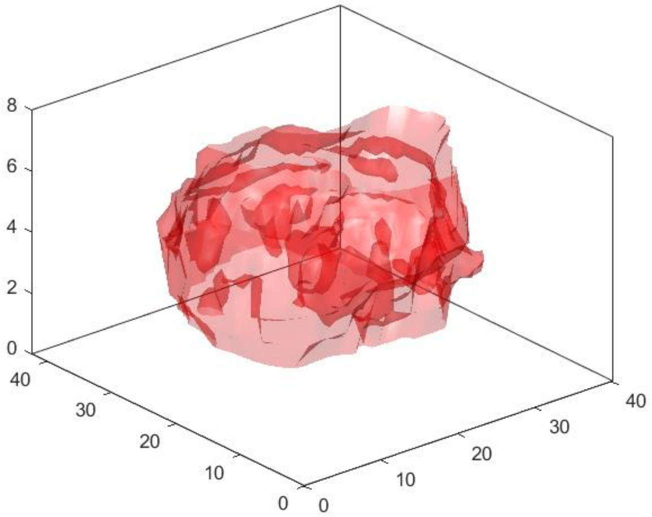

Supplementary S2. Detailed features of the radiomics analysis

Histogram analysis consisted of 14 features (energy, entropy, kurtosis, maximum, mean, mean absolute deviation, median, minimum, range, root mean square, skewness, standard deviation, uniformity, and variance). Shape- and size-based features consisted of 8 features (compactness 1, compactness 2, maximum 3D diameter, spherical disproportion, sphericity, surface area, surface to volume ratio, volume). GLCM features were analyzed for 22 parameters (autocorrelation, cluster prominence, cluster shade, cluster tendency, contrast, correlation, difference entropy, dissimilarity, energy, entropy, homogeneity 1, homogeneity 2, informational measure of correlation 1, informational measure of correlation 2, inverse difference moment normalized, inverse difference normalized, inverse variance, maximum probability, sum average, sum entropy, sum variance and variance). GLRLM features were analyzed for 11 parameters (short run emphasis, long run emphasis, gray-level non-uniformity, run-length non-uniformity, run percentage, low gray-level run emphasis, high gray-level run emphasis, short run low gray-level emphasis, short run high gray-level emphasis, long run low gray-level emphasis, and long run high gray-level emphasis).

Supplementary Table S3. Multivariable Cox proportional hazard regression performed with the best subset selection method in 6 models. Variables that were significantly associated with systemic recurrence on univariate analysis were selected for this analysis.

Model 1

Variable	Hazard ratio (95% CI)	*P* value
Pathologic invasive cancer size	1.049 (1.009, 1.09)	0.016
Rad score	34.401 (11.76, 100.629)	<0.001
Training C-index (95% CI)	0.96 (0.825, 1)	

Note.—CI = confidence interval

Model 2

Variable		Hazard ratio (95% CI)	*P* value
[*]pCR or not	Yes vs. No	0.01 (0, 0.287)	<0.001
	Not applicable vs. No	0.677 (0.231, 1.987)	0.465
Rad score		105.265 (21.133, 524.318)	<0.001
Training C-index (95% CI)		0.388 (0.253, 0.523)	

Note.—CI = confidence interval, pCR = pathologic complete response, [*]Unbiased hazard ratio was estimated with Firth’s penalized maximum likelihood estimation method because the data was completely separable.

Model 3

Variable		Hazard ratio (95% CI)	*P* value
Lymphovascular invasion	Yes vs. No	4.661 (1.619, 13.418)	0.004
Rad score		26.096 (10.356, 65.757)	<0.001
Training C-index (95% CI)		0.96 (0.825, 1)	

Note.—CI = confidence interval

Model 4

Variable	Hazard ratio (95% CI)	*P* value

*Histologic grade	2 vs. 1	0.196 (0.014, 2.705)	0.191
	3 vs. 1	0.171 (0.013, 2.302)	0.163
	Not available vs. 1	0.002 (0, 0.143)	0.001
Rad score		117.271 (24.221, 567.788)	<0.001
Training C-index (95% CI)		0.367 (0.232, 0.501)	

Note.—CI = confidence interval, *Unbiased hazard ratio was estimated with Firth's penalized maximum likelihood estimation method because the data was completely separable.

Model 5

Variable		Hazard ratio (95% CI)	*P* value
Surgery type	Breast conserving surgery vs. total mastectomy	0.269 (0.09, 0.808)	0.019
Rad score		29.067 (10.695, 78.997)	<0.001
Training C-index (95% CI)		0.966 (0.832, 1)	

Note.—CI = confidence interval

Model 6

Variable	Hazard ratio (95% CI)	*P* value
Number of metastatic axillary lymph nodes after surgery	1.043 (0.976, 1.115)	0.209
Rad score	23.02 (9.946, 53.282)	<0.001
Training C-index (95% CI)	0.965 (0.83, 1)	

Note.—CI = confidence interval
